# No Effect of Diet-Induced Mild Hyperhomocysteinemia on Vascular Methylating Capacity, Atherosclerosis Progression, and Specific Histone Methylation

**DOI:** 10.3390/nu12082182

**Published:** 2020-07-23

**Authors:** Courtney A. Whalen, Floyd J. Mattie, Cristina Florindo, Bertrand van Zelst, Neil K. Huang, Isabel Tavares de Almeida, Sandra G. Heil, Thomas Neuberger, A. Catharine Ross, Rita Castro

**Affiliations:** 1 Department of Nutritional Sciences, The Pennsylvania State University, University Park, PA 16802, USA; caw400@psu.edu (C.A.W.); fjm1311@gmail.com (F.J.M.); neil.huang@tufts.edu (N.K.H.); acr6@psu.edu (A.C.R.); 2 Faculty of Pharmacy, Universidade de Lisboa, 1649-003 Lisbon, Portugal; cristinaflorindo@ff.ulisboa.pt (C.F.); italmeida@ff.ulisboa.pt (I.T.d.A.); 3 Department of Clinical Chemistry, Medical Center Rotterdam, Erasmus MC University, 3015 GD Rotterdam, The Netherlands; b.vanzelst@erasmusmc.nl (B.v.Z.); s.heil@erasmusmc.nl (S.G.H.); 4 Cardiovascular Nutrition Laboratory, Jean Mayer USDA Human Nutrition Research Center on Aging, Tufts University, Boston, MA 02111, USA; 5 Huck Institutes of the Life Sciences, The Pennsylvania State University, University Park, PA 16802, USA; tun3@psu.edu; 6 Biomedical Engineering, The Pennsylvania State University, University Park, PA 16802, USA; 7 Research Institute for Medicines (iMed. ULisboa), Universidade de Lisboa, 1649-003 Lisbon, Portugal

**Keywords:** homocysteine and vascular disease, H3K27me3, epigenetics, atherosclerosis, MRI (magnetic resonance imaging)

## Abstract

Hyperhomocysteinemia (HHcy) is a risk factor for atherosclerosis through mechanisms which are still incompletely defined. One possible mechanism involves the hypomethylation of the nuclear histone proteins to favor the progression of atherosclerosis. In previous cell studies, hypomethylating stress decreased a specific epigenetic tag (the trimethylation of lysine 27 on histone H3, H3K27me3) to promote endothelial dysfunction and activation, i.e., an atherogenic phenotype. Here, we conducted a pilot study to investigate the impact of mild HHcy on vascular methylating index, atherosclerosis progression and H3K27me3 aortic content in apolipoprotein E-deficient (*ApoE* ^−/−^) mice. In two different sets of experiments, male mice were fed high-fat, low in methyl donors (HFLM), or control (HF) diets for 16 (Study A) or 12 (Study B) weeks. At multiple time points, plasma was collected for (1) quantification of total homocysteine (tHcy) by high-performance liquid chromatography; or (2) the methylation index of S-adenosylmethionine to S-adenosylhomocysteine (SAM:SAH ratio) by liquid chromatography tandem-mass spectrometry; or (3) a panel of inflammatory cytokines previously implicated in atherosclerosis by a multiplex assay. At the end point, aortas were collected and used to assess (1) the methylating index (SAM:SAH ratio); (2) the volume of aortic atherosclerotic plaque assessed by high field magnetic resonance imaging; and (3) the vascular content of H3K27me3 by immunohistochemistry. The results showed that, in both studies, HFLM-fed mice, but not those mice fed control diets, accumulated mildly elevated tHcy plasmatic concentrations. However, the pattern of changes in the inflammatory cytokines did not support a major difference in systemic inflammation between these groups. Accordingly, in both studies, no significant differences were detected for the aortic methylating index, plaque burden, and H3K27me3 vascular content between HF and HFLM-fed mice. Surprisingly however, a decreased plasma SAM: SAH was also observed, suggesting that the plasma compartment does not always reflect the vascular concentrations of these two metabolites, at least in this model. Mild HHcy in vivo was not be sufficient to induce vascular hypomethylating stress or the progression of atherosclerosis, suggesting that only higher accumulations of plasma tHcy will exhibit vascular toxicity and promote specific epigenetic dysregulation.

## 1. Introduction

Homocysteine (Hcy) is a sulfur-containing amino acid formed during the methionine metabolism. Mild hyperhomocysteinemia (HHcy), a condition defined by an accumulation of plasma tHcy between 15 and 25 µM, is highly prevalent in most Western populations, and may be an independent risk factor for atherosclerosis [1,2]. Nevertheless, the molecular basis of the association between Hcy and atherosclerosis is incompletely defined [1]; one possibility involves its impact on the cellular transmethylating reactions [3,4,5].

The intracellular concentration of Hcy is tightly regulated by several metabolic pathways, and it affects the cell methylating capacity, which is defined as the ratio of S-adenosylmethionine (SAM) to S-adenosylhomocysteine (SAH) in methionine metabolism [2,5]. SAM is the methyl donor to several methyltransferases that target innumerous biomolecules, including DNA and proteins. Importantly, excess SAH inhibits the activities of these SAM-dependent methyltransferases, which results in decreases in the SAM:SAH ratio and intracellular methylating capacity [6,7]. SAH is further converted into Hcy by a reaction that is reversible and strongly favors SAH synthesis rather than hydrolysis. Thus, if Hcy accumulates, SAH accumulates as well, decreasing the SAM: SAH ratio and causing hypomethylating stress [3,8,9].

Several observations from in vitro and in vivo studies support the concept that hypomethylating stress contributes to the adverse vascular consequences of HHcy [1,2,3,6,7,8,9,10,11,12,13]. This possibility has been also supported by several human studies. For example, one recent cohort study found that higher plasma SAH concentrations were positively associated with cardiovascular disease (CVD) risk in patients undergoing coronary angiography [14]. In another study, plasma concentration of SAH, but not of Hcy, was strongly associated with traditional CVD risk factors and subclinical atherosclerosis in subjects at low CVD risk [15]. Moreover, plasma SAH was found to be inversely associated with a measure of endothelial dysfunction (namely, endothelial nitric oxide synthase-dependent vasodilation) in a small sample of patients with coronary artery disease [16].

Several cell studies have documented the hypomethylating effect associated with decreased SAM: SAH ratios on epigenetically relevant targets that may contribute to the adverse vascular effects of Hcy accumulation [3,6,7]. Epigenetic modulators of gene expression include histone methylation [4]. Our prior study reported that hypomethylating stress contributes to a proatherogenic environment by down-regulating the activity of the SAM-dependent histone lysine methyltransferase known as an enhancer of zeste homolog 2 (EZH2) [17]. EZH2 silences gene expression by catalyzing the tri-methylation of histone H3 at lysine 27 (H3K27me3). EZH2 was identified as a new target affected by a hypomethylating environment, which caused a decrease in the endothelial content of H3K27me3 [17], promoting a pro-atherogenic phenotype [2,17]. Consistent with the involvement of this specific epigenetic modification in atherosclerosis progression, a reduction in H3K27me3 content in human plaques has been reported in two studies [18,19].

As aforementioned, mild HHcy is a condition highly prevalent in most populations and an independent risk factor for atherosclerosis by mechanisms which are incompletely defined. In light of these observations, we investigated whether a mild Hcy accumulation in vivo induces alterations in vascular methylating index and in the epigenetic marker, H3K27me3, to favor atherosclerosis progression. The remethylation of Hcy to methionine requires folate and vitamin B12 (cobalamin) as co-factors. Alternatively, Hcy can be remethylated to methionine by betaine, which is a choline metabolite. Moreover, the catabolism of Hcy to cysteine requires vitamin B6. Thus, dietary manipulation of the content of methyl donors and B vitamins is an established approach to produce the accumulation of tHcy in mice, especially in the presence of an excess of methionine [12,20,21]. With this purpose, we fed genetically susceptible apolipoprotein E-deficient (*ApoE* ^−/−^) mice [22] a high-fat diet hypomethylating (HFLM) diet, or a high-fat control diet (HF) with a normal content of methyl donors. After confirming the presence of mild HHcy only in the HFLM-fed mice, we measured systemic inflammation, the plasma and aortic SAM:SAH ratio, and compared the extent of atherosclerotic lesions formation. The volume of atherosclerosis in the aortas was quantified using advanced imaging techniques (high resolution magnetic resonance imaging, MRI). Lastly, following MRI analysis, the scanned aortas were subjected to the quantification of the epigenetic tag H3K27me3.

## 2. Methods

### 2.1. Animals and Diets

Seven-week-old *ApoE* ^−/−^ mice (Jackson Laboratory, Bar Harbor, ME, USA) were housed individually in cages with wire grid floors and fed diets that contained 1% sulfathiazole to ensure that the animal’s only source of B vitamins was from the diet. Only male mice were included, to control for the known effect of gender on atherosclerosis in this strain [23]. The animals were maintained in a room at 22 ± 2 °C with a 12-h light–dark cycle with free access to water. Mice were fed ad libitum for 16 weeks (Study A) or 12 weeks (Study B), with one of the following diets prepared based on AIN 93M (Research Diets, New Brunswick, NJ, USA) [12,20]: a control diet (C, 11 Kcal% fat, 70 Kcal% carbohydrate, 19 Kcal% protein), an HF diet (HF, 40 Kcal% fat, 41 Kcal% carbohydrate, 19 Kcal% protein and 0.15% cholesterol), or an HFLM diet enriched in methionine and with decreased levels of methyl donors and vitamins (folate, choline, vitamin B6, and vitamin B12), as previously described [12,20]. The composition of the experimental diets is shown in the supplementary Table S1. Diets were replaced once a week, at which point animals and the remaining food were weighed. Food consumption was estimated as an average per mouse per day within each cage. All procedures were performed in compliance with the Institutional Animal Care and Use Committee of the Pennsylvania State University, which specifically approved this study.

### 2.2. Blood Collection

After mice were fed for 4, 8 and 12 weeks on each diet, blood was collected from the retroorbital cavity into heparinized tubes and immediately placed on ice. Plasma was isolated by centrifugation at 4 °C and immediately stored at −80 °C prior to further biochemical analyses.

### 2.3. Aorta Collection and Preparation of Lysates

After 16 (Study A) or 12 weeks (Study B) on each diet, mice were euthanized by carbon dioxide inhalation and, after the exposure of the aorta, 10 mL of cold phosphate saline buffer (PBS) was perfused using a syringe through the left ventricle of the heart. Approximately one third of the descending thoracic aorta was excised, transferred into 100 µL of a solution of acetonitrile, methanol, and water (2:2:1) containing 0.1M HCl (to preserve SAH during the subsequent lysate preparation), immediately immersed into liquid nitrogen, and stored at −80 °C until the preparation of the lysates for SAM and SAH quantification, described below. The remainders of the aorta (ascending aorta, aortic arch, and remaining descending thoracic aorta) was then perfused through the heart in situ via the left ventricle with 10% neutral buffered formalin (NBF) in PBS, and dissected from other tissues. The aortic tissue was fixed in 10% NBF overnight, washed with PBS, and subjected to Oil Red O staining, as described below.

Aorta lysates for SAM and SAH quantification were subsequently prepared by homogenization with 2.8 mm ceramic beads using a Bead Ruptor 12 (Omni, Kennesaw, GA, USA) with two cycles of 15 s at the maximum speed, with 30 s on ice in between. The lysate was cleared by centrifugation at 12,000 *g* and 4 °C for 10 min and immediately stored at −80 °C until SAH and SAM analysis.

### 2.4. Homocysteine and Methylating Index (SAM:SAH Ratio)

Plasma total Hcy (tHcy), defined as the total concentration of Hcy after the reductive cleavage of all disulfide bonds, was determined by high-performance liquid chromatography (HPLC) analysis according to an adapted method of Araki and Sako [24], and as previously described [11].

SAM and SAH levels were measured in plasma (after 8 weeks on each diet) and in aortic lysates (at the end point) using a liquid chromatography tandem-mass spectrometry (LC-MS/MS) method after the use of solid-phase extraction columns according to an adapted method of Gellekink et al. [25], and as previously described [26]. The stability of SAM and SAH in plasma was shown to be 100% if samples were stored immediately at −80 °C [23]. As aforementioned, to protect aortic lysates from degradation of SAM, we acidified the aortic lysates with 0.1M HCl, after which samples were stored at −80 °C [25]. Within-run precision was shown to be 2.9% and 2.4% for SAM and SAH, respectively.

### 2.5. Systemic Inflammation

Interleukin 6 (IL-6), interleukin 10 (IL-10), monocyte chemoattractant protein 1 (MCP-1), and tumor necrosis factor (TNF-α), previously identified in the pathogenesis of atherosclerosis [3], were measured in duplicate at two different time points (4 weeks and 12 weeks) in Study A, using MSD U-PLEX multiplex assay platforms (Meso Scale Diagnostics, Rockville, MD, U.S.) following the manufacturer’s instructions.

### 2.6. Aorta Processing for MRI Analysis

Fixed aortas were stained with Oil Red O (EMD chemicals, Cat. #3125-12) to facilitate complete adventitial fat removal during dissection. Briefly, aortas were dehydrated in successive methanol–water solutions (25%, 50%, 78% methanol for 15 min each at room temperature, RT, with rocking). Aortas were then incubated in Oil Red O working solution (5 parts 2.8% Oil Red O in methanol: 2 parts 1 M sodium hydroxide) for 2 h at RT, with rocking. Subsequently, aortas were washed and rehydrated with successive PBS solutions with the following methanol concentrations (%): 78, 50, 25, or 0 (15 min each solution, RT, rocking). Connective tissues surrounding the aortas were dissected away under a stereomicroscope. Dissected aortas were then equilibrated in a solution of 0.1% Magnevist (Bayer), 0.25% sodium azide in PBS overnight at 4 °C, then placed into glass tubes (6 mm OD, 4 mm ID, 60 mmL) for MRI analysis.

### 2.7. MRI Analysis: Atherosclerotic Plaque Volume

Aortic plaque burden was determined by MRI, as previously described, but using an Agilent 14T micro imaging system and a home-built saddle coil with an inner diameter of 7 mm [27,28,29]. A previously validated [30] gradient echo imaging sequence with an imaging time of 9 h 48 min was used to generate 3D datasets of the aortas. Scan parameters included an echo time (TE) of 13 ms, a repetition time (TR) of 100.00 ms, eight averages, a field of view (FOV) of 12.6 × 4.2 × 4.2 mm^3^ and a matrix size of 630 × 210 × 210, resulting in an isotropic resolution of 20 µm. After acquisition, MR data was reconstructed using Matlab (The MathWorks Inc., Natick, MA, USA). Zero-filling by a factor of 2 in each direction lead to a final isotropic pixel resolution of 10 µm.

Data segmentation was performed using Avizo 9.5 (Thermo Fisher Scientific, Waltham, MA, USA). The lumen of the aorta, the different plaques, and the aorta wall were manually segmented using the lasso tool. Quantification of plaque volume was determined using the material statistics function in Avizo 9.5 on the segmented aorta. The results were expressed as the percent of plaque area in relation to the total segmented area (plaque + lumen + wall).

### 2.8. Aorta Cryosectioning and Immunofluorescence Analysis: Specific Histone Methylation

**Figure 1:**
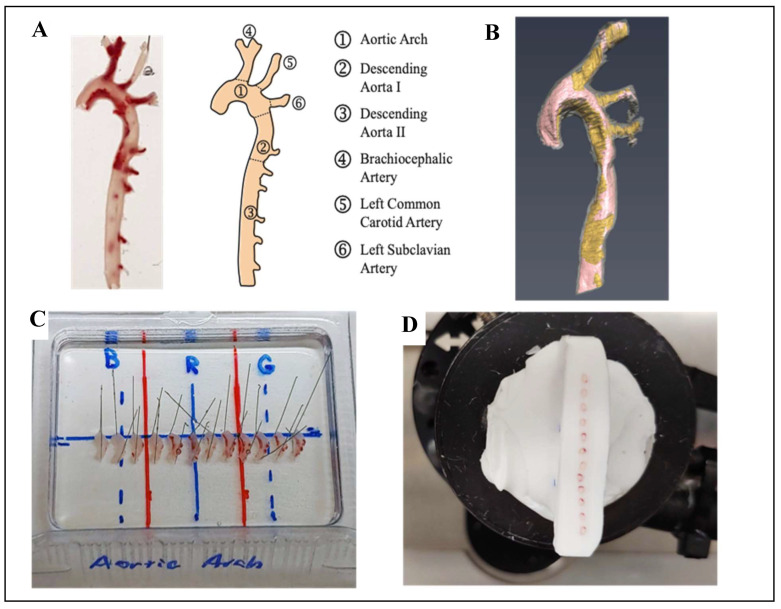
Aorta Cryosectioning (**A**) Example of one aorta from *ApoE* ^−/−^ mice and schematic representation of the different regions of the aorta including pre-embedding cut sites (dashed lines); (**B**) Example of the visualization of the aortic arteriosclerotic plaque (colored in yellow) using 14T-magnetic resonance imaging (MRI); (**C**) After MRI analysis, aortas from the same study were cut, each region was pinned into alignment, embedded in Optimal Cutting Temperature (OCT) compound and stored at −20 °C for 24 h. Blue letters B, R, and G only represent marks used as orientation and spacing guides. (**D**) The resulting cryoblock was then sectioned, creating arrays of aortic slices of similar orientation and position for immunofluorescence analysis.

Following MRI analysis, aortas were equilibrated in sucrose solution. Aortas were then cut into four regions, pinned out into arrays, embedded in Tissue-Tek Optimal Cutting Temperature compound (OCT, Sakura Finetek, Tokyo, Japan) and stored at −20 °C (Figure 1). Aorta arrays were sectioned at an angle generally perpendicular to the vessel into 12 µm thick sections using a Leica CM3050 S cryostat, at −20 °C, and attached to TruBond 380 microscope slides (Electron Microscopy Sciences, Hatfield, PA, USA). Slides were dried at least 1 h at 32 °C, then stored at −20 °C.

Heat-Induced Epitope Retrieval (HIER) was conducted for 30 min at < 100 °C in a decloaking chamber (Biocare Medical, Pacheco, CA, USA), setting 9.5, using Rodent Decloaker solution (Biocare Medical, Pacheco, CA, USA) in accordance with manufacturer-provided protocols. Arrays were then encircled with an ImmEdge Hydropphobic Barrier PAP pen (Vector Laboratories, Burlingame, CA, USA), blocked with 2% BSA, 1% NGS, 0.05% Triton X-100, 0.05% Tween-20, 0.05% sodium azide in PBS (blocking buffer) for ≥ 1 h, and then incubated with primary antibodies in blocking buffer overnight at 4 °C. After washing, arrays were incubated with secondary antibodies in blocking buffer for ≥ 1 h at RT, washed, treated with Vector TrueVIEW Autofluorescence Quenching Kit (Vector Laboratories, Burlingame, CA, USA), and mounted under coverslips in VECTASHIELD Vibrance Antifade Mounting Medium (Vector Laboratories). The antibodies used were: rabbit anti-histone H3K27me3 pAb (1:200, Cat.# A-4039, Epigentek, Farmingdale, NY, USA, Cat.# A-4039), mouse anti-histone H3 mAb 1B1-B2 (1:200, Cat.#819404, BioLegend, San Diego, CA, USA), Alexa Fluor 555 goat anti-rabbit IgG (1:250, Cat.# A-21428 Invitrogen, Carlsbad, CA, USA), Alexa Fluor 488 goat anti-mouse IgG3 (1:250, Cat.# A-21151, Invitrogen, Carlsbad, CA, USA). The residual Oil Red O in the slides did not interfere with the immunofluorescence detection (Figure S1), likely due to HIER or time since Oil Red O staining [31].

Immunofluorescence images were collected using an Olympus BX61 widefield fluorescence microscope at the Penn State Microscopy Facility (University Park, PA, USA). Images were segmented and quantified using FIJI [32]. Briefly, the aortic lumen, plaque, and outside of the aorta were segmented manually as Regions of Interest (ROIs). All other ROIs were calculated through additions of and/or subtractions from these initial segments. Histone H3 fluorescence images were used to define nuclei within these ROIs and fluorescence within these nuclei selections were measured in both the histone H3 and histone H3K27me3 immunofluorescence images. Autofluorescence from the elastic fibers within the vessel walls was suppressed using TrueVIEW^®^ Autofluorescence Quenching Kit from Vector Laboratories. This allowed selection of the nuclei based on thresholding of the histone H3 fluorescence channel (green, Alexa488) image. Then, using only those areas selected as nuclei, the fluorescence of both the H3 (green, Alexa488) and H3K27me3 (red, Alexa555) channels could be quantified. Average fluorescence values from the nuclei contained in the various ROIs were collected for each aortic/vessel image. Average fluorescence intensity values within the nuclei represent the average pixel value over the whole group of selected nuclei (not the average intensity per nuclei). Those average fluorescence values were used to calculate the ratio of H3K27me3 to H3, which was used to assess relative changes in H3K27me3 content.

### 2.9. Statistical Analysis

Analyses were performed in GraphPad Prism 7 (GraphPad Software, La Jolla, CA, USA), with statistical significance set to *p*< 0.05. For comparison of two groups, an unpaired Student’s *t*-test was used. For more than two groups, a one- or two-way analysis of variance (ANOVA) was performed and adjusted for multiple comparisons.

## 3. Results

### 3.1. General Characteristics

**Figure 2:**
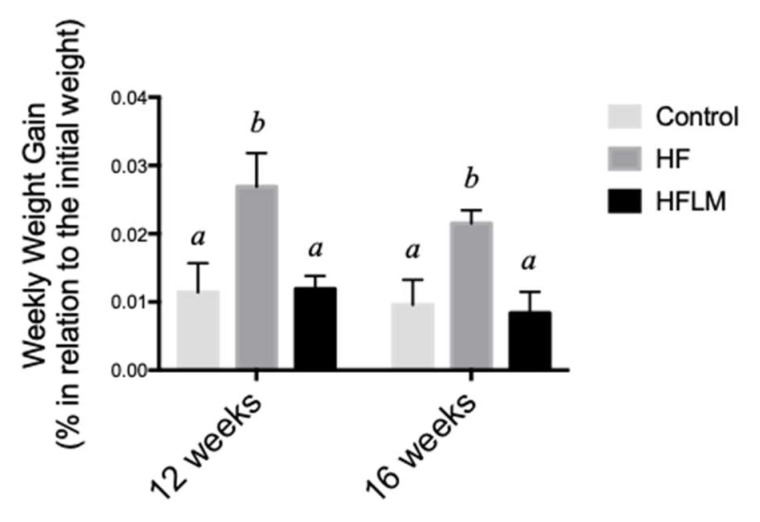
The effect of control, High-Fat (HF), or low in methyl donors (HFLM) diets on growth. Results are weekly weight gain (% of initial weight). Data shown are the mean ± SEM; *n* = 5–6/group, bars not sharing superscript letters differ from each other, *p* < 0.05.

The mean food per week did not differ between HF and HFLM groups (data not shown). Nevertheless, after 16 weeks (Study A), HF mice gained more weight than HFLM mice (*p* < 0.05) or control mice (*p* < 0.05), while the latter two groups did not differ from each other (Figure 2). To exclude the causal role of secondary metabolic effects caused by an extended period of dietary treatment with suboptimal levels of micronutrients, a second group of mice was fed for a shorter period (12 weeks, Study B). Again, the HFLM-fed mice grew more poorly (*p* < 0.05) than the other two groups of mice.

### 3.2. Homocysteine and Methylating Index (SAM:SAH Ratio)

**Figure 3:**
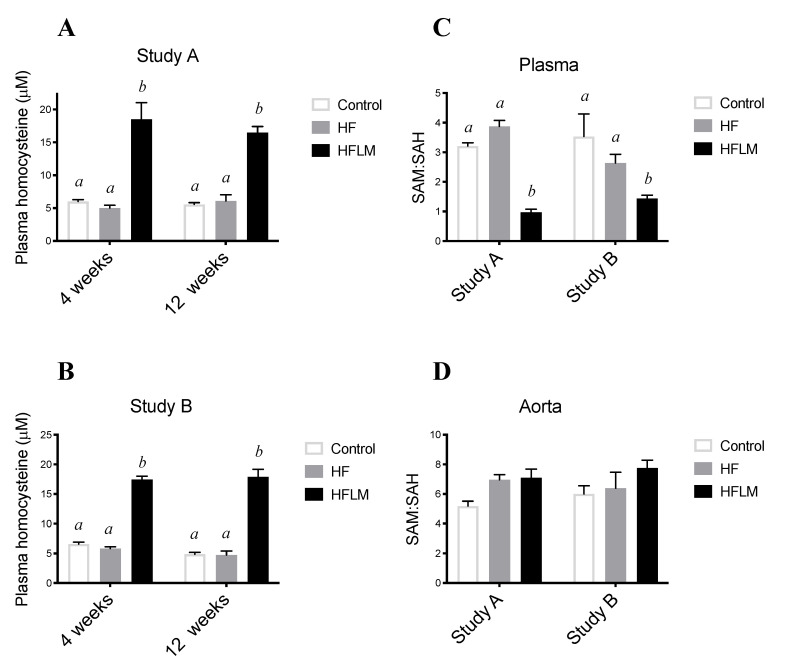
The effect of control, High-Fat (HF), or low in methyl donors (HFLM) diets on plasma tHcy concentrations (**A**,**B**), plasma SAM:SAH ratio (**C**), and aortic SAM:SAH ratio (**D**). Data shown are the mean ± SEM, *n* = 5–6/group; bars not sharing not sharing superscript letters differ by *p* < 0.05.

Mild HHcy was induced by the HFLM diet in both studies (A and B) (Figure 3) (*n* = 5–6 per study). In fact, in study A, and at week 4 and week 12, HFLB-fed mice had higher (*p* < 0.05) plasma tHcy concentration when compared to the HF-fed mice or control-fed mice (Figure 3A). These observations were further confirmed in study B, in which, equally after 4 or 12 weeks, HFLM-fed mice, but not those mice fed HF or control diets, accumulated mildly elevated tHcy plasmatic concentrations (25 µM > tHcy > 15 µM) (Figure 3B).

The ratio of SAM concentration divided by SAH concentration was used as an indicator of the methylating capacity, determined in plasma after 8 weeks of diet treatment, and in the aortas after 16 weeks (Study A) or 12 weeks (Study B) of diet treatment. In both studies (*n* = 5–6 per study), HFLM mice displayed a significantly lower plasma SAM: SAH ratio, when compared to HF or control mice (Figure 3C). In both studies, plasma SAM: SAH ratios were similar in HF and control mice (Figure 3C). Interestingly, however, the ratio of aortic concentrations of SAM: SAH in HFLM mice did not differ from HF or control mice either after 12 weeks or 16 weeks of diet (Figure 3D), thus showing the absence of an hypomethylating environment in the HFLM aortas.

### 3.3. Systemic Inflammation

**Figure 4:**
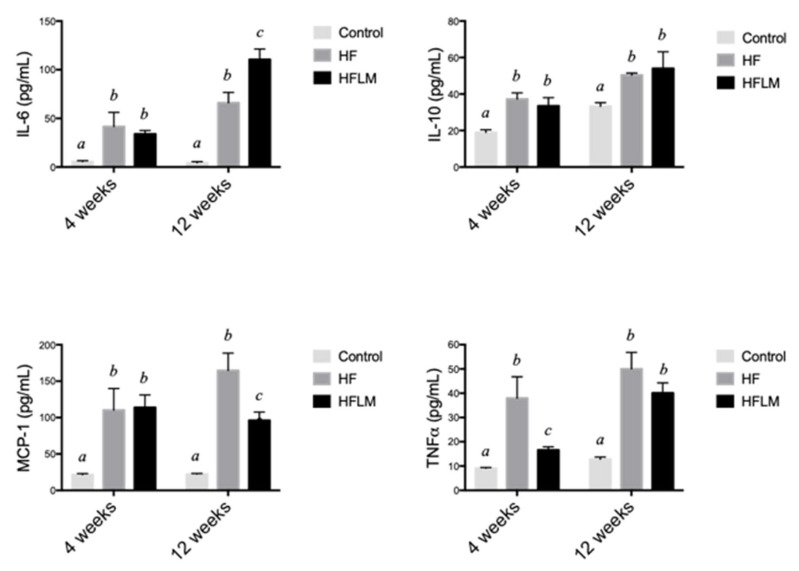
Systemic concentrations of interleukin 6 (IL-6), interleukin 10 (IL-10), monocyte chemoattractant protein 1 (MCP1) and tumor necrosis factor α (TNF- α) in mice fed- low in methyl donors (HFLM) diet, High-Fat (HF) diet or control diet during 16 weeks (study A). Results are mean ± SEM (*n* = 4 per group). Data not sharing superscript letters differ by *p* < 0.05.

Cytokines were measured at two different time points (4 weeks and 12 weeks) in Study A to evaluate the status of systemic inflammation, a key component of the atherosclerosis process. At both times, IL-6, IL-10, MCP1, and TNF α concentrations were significantly lower in control mice compared to mice fed either the HF or HFLB diet (Figure 4). Apart from this, mice fed HF diet, when compared at the same time to mice fed HFLM diet, had similar levels of all cytokines with few exceptions: TNF α was significantly elevated in HF mice at 4 weeks (but was similar after 12 weeks of diet); IL6 was elevated in the HFLB mice after 12 weeks; MCP-1 was higher in HF mice after 12 weeks (Figure 4).

### 3.4. Atherosclerotic Plaque Volume

**Figure 5:**
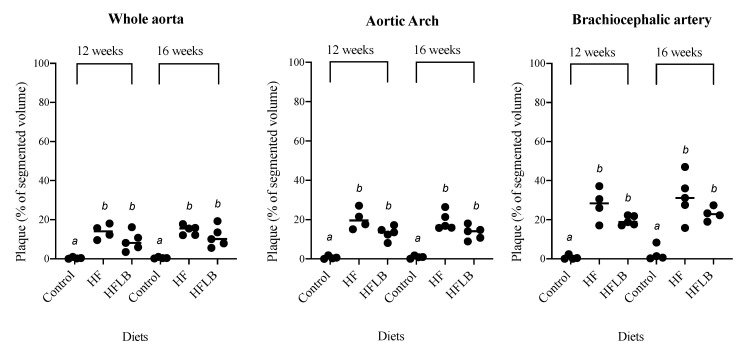
Ex vivo 14T-MRI volumetric assessment of the aortic atherosclerotic plaque in mice fed-hypomethylating (HFLM) and High-Fat (HF) diets, for 12 or 16 weeks. Plaque denotes the volume of plaque relative to the total segmented area. Data not sharing superscript letters differ by *p* < 0.05. Black circles represent individual values and horizontal lines represent the mean value for each group.

A method based on ex vivo MR imaging was used to visualize and quantify the volume of atherosclerotic plaque. Distribution of the plaque was similar in all three diets, predominating in the aortic arch and BCA. Plaque volume was significantly increased in HF and HFLM groups compared to the control group, but no significant differences were detected (at the *p* < 0.05 level), between HF and HFLM groups, at either 16 weeks (Study A) or 12 weeks (Study B) (Figure 5). Moreover, this observation was independent of the segment of aorta analyzed (shown in Figure 1A): ascending aorta, aortic arch, or descending thoracic aorta. When mice fed the same diets were compared, there was a consistent increase in plaque burden over time, from 12 to 16 weeks, although the magnitude differed between mice fed the control and mice fed either of the HF diets.

### 3.5. Specific Histone Methylation

**Figure 6:**
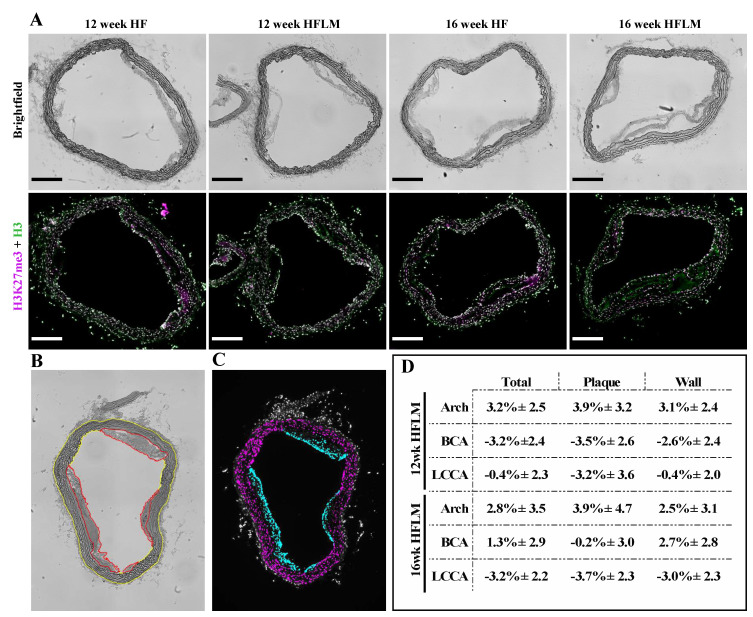
Immunofluorescent (IF) analysis (**A**) Brightfield and IF images of aortic arch cross sections from *ApoE* ^−/−^ mice fed either a High Fat (HF) or a High Fat Low Methyl donors (HFLM) diet for 12 or 16 weeks (wk) as indicated. Total Histone 3 (H3) in green, and Histone 3 trimethylated at lysine 27 (H3K27me3) in magenta. Scale bars = 200 μm. (**B**) Example of the manual segmentation using FIJI (ImageJ) to define Plaque (Red) and Wall (Yellow); (**C**) Example of the Plaque Nuclei (Cyan) and Wall Nuclei (Magenta) segments used to measure mean fluorescence ratios of H3K27me3:H3; (**D**) Variation of the relative H3K27me3 content in the HFLM aortas in relation to the corresponding HF aortas expressed as percent change; data are the mean ± SEM, *n =* 25. BCA, brachiocephalic artery; LCCA, left common carotid artery (localization shown in Figure 1A).

Immunofluorescence was used to evaluate the content of H3K27me3 (in relation to total histone H3) in the HFLM aortas compared to the HF aortas (Figure 6), after 12 or 16 weeks of mild HHcy. Different aortic segments (aortic arch; brachiocephalic artery, BCA); and left common carotid artery, LCCA), and different ROIs (total vessel, plaque, and wall) were considered and, as shown in Figure 6D, no significant differences were detected (at the *p* < 0.05 level), for the content of the epigenetic tag H3K27me3.

## 4. Discussion

The present study investigated in an in vivo model of atherogenesis whether mild HHcy alters aortic methylating index and the vascular epigenetic content of H3K27me3 to favor a pro-atherogenic phenotype.

SAH is the precursor of Hcy that may accumulate in the setting of HHcy to cause hypomethylating stress, and this, in turn, may contribute to the vascular toxicity [2,3,6,7]. This possibility is based on the ability of SAH, once its intracellular concentration reaches a certain threshold, to inhibit the methyltransferase reactions that rely on SAM. EZH2 is a SAM-dependent methyltransferase that participates in histone methylation and chromatin compaction, negatively regulating gene expression [33]. EZH2 establishes the epigenetic tag H3K27me3 that represses the expression of genes responsible for the establishment of a proatherogenic endothelial phenotype [34]. Accordingly, we have previously shown that, in endothelial cells, hypomethylating stress decreases H3K27me3 to promote inflammation and endothelial dysfunction and activation [17]. However, the in vivo effect of mild HHcy on the vascular methylating index and content of this epigenetic tag is elusive.

Dietary manipulation of the content of methyl donors and B vitamins is an established approach to produce the accumulation of tHcy in mice, especially in the presence of an excess of methionine [12,20,21]. Accordingly, in the present study, a mild but significant accumulation of circulating tHcy was achieved in the mice fed the HFLM diet [35].

Many studies have used dietary approaches similar to ours in *ApoE* ^−/−^ mice to explore the vascular phenotype associated with HHcy. Studies in which a moderate (25–50 µM) or severe (> 100 µM) accumulation of plasma tHcy was achieved reported that HHcy enhanced the development of atherosclerosis [21]. However, the findings associated with mild HHcy are inconsistent. For example, Liu et al. [36] reported that 8 weeks of severe HHcy with a concomitant decrease in SAM:SAH resulted in aggravated aortic plaque formation; however, a milder elevation of Hcy that did not alter plasma SAM:SAH did not alter the extent of atherosclerotic plaque development. More recently, Xiaoling et al. [37] reported that a similar mild elevation of Hcy increased aortic lesion area after 16 weeks of diet using a high-fat diet enriched in methionine, but no measures of methylating status were provided in this report. Interestingly, in a genetic model of mild HHcy achieved by crosses of *ApoE* ^−/−^ mice, with mice having a genetic defect in Hcy metabolism (cystathionine beta-synthase, CBS, deficiency), *ApoE* ^−/−^ *CBS* ^−/−^ mice developed more severe aortic root lesions than *ApoE^−/−^* mice at 12 months of age but not at earlier timepoints of 15 weeks or 6 months [38]. In the present study, a mildly elevated plasma Hcy for 16 weeks (Study A) (Figure 3A) did not significantly affect atherosclerosis progression (Figure 5) or aortic SAM: SAH values (Figure 3C). To the best of our knowledge, no previous murine studies have provided measures of the vascular methylating index under mild HHcy. Surprisingly however, a decreased plasma SAM: SAH, an indicator of systemic hypomethylating status, was also observed after 8 weeks of diet (Figure 3D), suggesting that the plasma compartment does not always reflect the vascular concentrations of these two metabolites. To confirm these observations, and to exclude the causal role of secondary metabolic effects due to an extended period of dietary treatment with suboptimal levels of micronutrients, a second group of mice was subjected to the same experimental design but for a shorter period (12 weeks, Study B). Again, plasma Hcy concentrations were in the range of mild HHcy (Figure 3B) and were not associated with aortic hypomethylation (Figure 3D) nor atherosclerosis progression (Figure 5). Moreover, and similarly to study A, lower plasma SAM:SAH was also observed in study B in HFLM mice when compared to both other groups (Figure 3C). In fact, it is known that methylation-regulating enzymes are differentially expressed in different murine tissues, which results in tissue-specific differences in SAM and SAH concentrations [12,13]. Our results suggest that, in this model, under mild HHcy, the systemic SAM:SAH ratio does not always reflect the vascular methylating capacity.

In the present study, ex vivo 14T-MR imaging was used to quantify the extent of atherosclerosis in the aortas of *ApoE* ^−/−^ mice enabling the visualization, and subsequent quantification of total plaque volume with sensitivity [27,28,29]. Moreover, this method preserved the aortic tissue that was further used to quantify histone methylation. Our observation that the volume of vascular lesions over the entire aorta was not higher in the hyperhomocysteinemic mice (HFLM) was further confirmed by evaluating plaque volume in segments of aorta that are more prone to develop atherosclerosis (BCA and aortic arch) (Figure 5). In these highly susceptible regions, no significant differences in the plaque burden were detected between HF and HFLM mice. Importantly, however, HFLM mice grew more slowly than HF animals, and this may have retarded the plaque progression in these animals explaining the observed absence of vascular toxicity. Previous studies have also noted a significant decrease in body weight in animals fed diets with high methionine and a lack of micronutrients [20,21]. Lastly, as anticipated, plaque volume in HF and HFLM mice was significantly increased compared to that in control mice (Figure 5), indicating a role of high-fat diet, regardless of B vitamin content, in the development of atherosclerosis.

The effect of diet on the circulating levels of cytokines previously implicated in the pathogenesis of atherosclerosis was also investigated previously [39]. IL-6 is an inflammatory cytokine that plays a central role in propagating the downstream inflammatory response that sustains atherosclerosis progression [40], whereas IL-10 is a prototypic anti-inflammatory cytokine released by activated macrophages that delays the growth of vascular lesions [41]. MCP1 is deeply implicated in the infiltration of immune cells into the vessel wall, an essential component of atherosclerosis progression [42]. Lastly, TNF-α is a key cytokine that facilitates the influx of inflammatory cells into the vessel wall, thus promoting the progression of the vascular lesions [43]. In the present study, both HF and HFLM diets promoted a significant systemic elevation of all the four cytokines, when compared to the control diet (Figure 4). This observation is in agreement with the positive effect of dietary fat on systemic inflammation [44]. Moreover, the increased systemic inflammation in HF and HFLM mice is consistent with the augmented arteriosclerotic plaque burden that was observed in these groups compared to control mice. Although some significant differences in plasma cytokines were present between HF and HFLM mice (Figure 4), the pattern of these changes does not support a major difference in disease mechanisms between these groups. Rather, the cytokine findings suggest comparable systemic inflammation in both the HF and HFLM groups, which is consistent with the plaque burden observed (Figure 5). Nevertheless, considering the systemic hypomethylation detected in the hyperhomocysteinemic animals, we are tempted to speculate that the up-regulation of IL6 expression observed in these animals after 12 weeks of diet when compared to the HF-fed mice (Figure 4) may result from the demethylation of the CpG islands in the promoter region of the gene encoding for this inflammatory cytokine [45,46]. In fact, DNA hypomethylation was previously observed under hypomethylating stress [3,4,8,9] and the decrease in promoter methylation status is a widely described mechanism associated with increased gene expression levels [3].

The ability of excess Hcy to disturb specific histone methylation has been demonstrated previously in murine models. Previously, we found that diet-induced moderate HHcy in rats decreased dimethylation of arginine 3 on histone H4 in a tissue-dependent manner [12]; however, in this study not all tissues analyzed showed a change in SAM: SAH ratio. Moreover, a severe accumulation of Hcy due to a genetic defect in Hcy metabolism (CBS deficiency) lessened the methylation of histone H3 at the arginine 8 position in the liver [13]. In a study in which the effect of excess SAH in *ApoE* ^−/−^ mice was explored in the absence of excess Hcy, plasma SAH was positively associated with increased atherosclerosis and negatively associated with the content of trimethylated histone H3 on lysine 9 in aortic lesions [47]. More recently, *ApoE* ^−/−^ mice were challenged with a high-fat high-methionine diet for 16 weeks to cause mild HHcy and, surprisingly, these HHcy mice had increased aortic levels of H3K27me3 [37], however, the SAM: SAH ratio was not measured in this model. As aforementioned, the epigenetic tag H3K27me3 is established by the SAM-dependent methyltransferase, EZH2, the activity of which is decreased by excess SAH, at least in endothelial cells under pharmacological treatments that cause a 6-fold reduction in the SAM: SAH ratio [17]. Thus, this paradoxical observation of increased levels of H3K27me3 under mild HHcy led us to investigate whether, in the aortas of our animals with mild HHcy, the same epigenetic tag was affected. To do so, we used a systematic immunofluorescence approach which revealed comparable amounts of this epigenetic tag in the HFLM and HF groups (Figure 6). This finding is consistent with the maintenance of the cell-methylating index in the aortas of HFLM mice when compared to HF mice, suggesting that, in this model under mild plasma HHcy, the concentrations of SAH accumulated in the tissue are below the Ki value for EZH2 [48].

## 5. Conclusions

In conclusion, in the present pilot study, two different sets of experiments were conducted, leading to the same observations: mildly elevated Hcy was associated with systemic hypomethylation but no differences between HLLM- and HF fed-mice were detected in vascular methylation status, specific epigenetic content and atherosclerosis progression. However, we do acknowledge that confirmatory studies with a higher number of animals may be needed to confirm these intriguing observations. Moreover, additional studies are being conducted using a diet promoting a more severe accumulation of Hcy to investigate the involvement of this phenotype on atherosclerosis progression via specific epigenetic dysregulation.

## Supplementary Materials

The following are available online at https://www.mdpi.com/2072-6643/12/8/2182/s1, Table S1. Composition of the experimental diets (Control; HF, High Fat; HFLM, High Fat Low Methyl). Figure S1. Lack of fluorescence from Oil Red O post processing for IF detection.
