# Supplementary material for: No Effect of Diet-Induced Mild Hyperhomocysteinemia on Vascular Methylating Capacity, Atherosclerosis Progression, and Specific Histone Methylation
Source: Nutrients. 2020 Jul 23;12(8):2182. doi: 10.3390/nu12082182 (PMC7468910; doi:10.3390/nu12082182)
Supplement: Supplementary file 1 [file nutrients-12-02182-s001.pdf]

Table S1. Composition of the experimental diets (Control; HF, High Fat; HFLM, High Fat Low Methyl).

**Ingredient**	**Control**		HF		HFLM	
	**gm**	**Kcal**	gm	Kcal	gm	Kcal
Casein	180	720	184	736	184	736
Corn Starch	431	1725	217	868	216	866
Maltodextrin 10	155	620	93	372	93	372
Sucrose	100	400	102	408	102	408
Cellulose	35	0	35	0	35	0
Cocoa Butter	0	0	155	1395	155	1395
Primex	25	225	0	0	0	0
Corn Oil	25	225	25	225	25	225
Mineral Mix S1000	35	0	35	0	35	0
Vitamin Mix V10001	10	40	10	40	0	0
Vitamin Mix V14904[1]	0	0	0	0	10	40
L-Cystine	3	12	3	12	3	12
L-Methionine	0	0	0	0	3.2	13
Choline bitartrate	2.5	0	2.5	0	0	0
Cholesterol	0	0	11	0	11	0
Pyridoxine HCl (× 10^3)	0	0	0	0	0.2	0
Folic acid (× 10^3)	0	0	0	0	0.1	0
Cyanocobalamin, 0.1% (× 10^6)	0	0	0	0	2	0
Succinylsulfathiazole	8.6	0	8.6	0	8.6	0

[1] Without vitamin B6, B9 or B12.

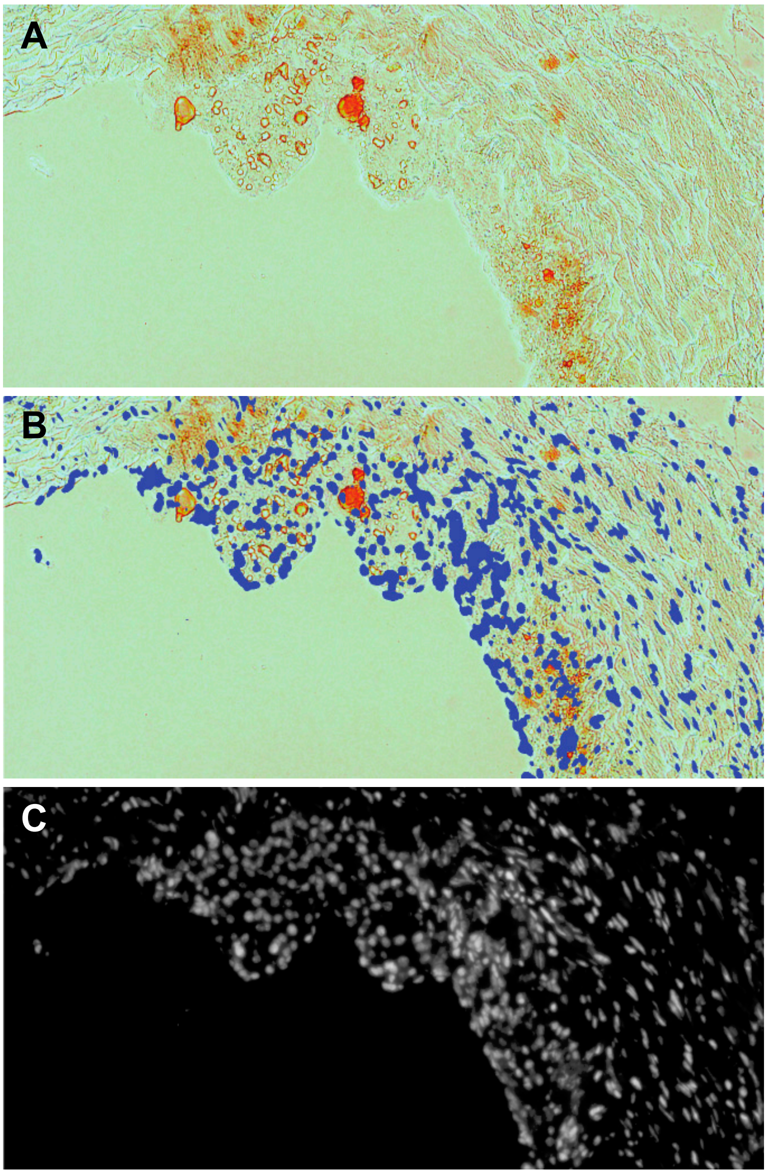

Figure S1. Lack of fluorescence from Oil Red O post processing for IF detection. As Oil Red O has been shown to have fluorescence [31], we needed to ensure that residual Oil Red O in our slides was not interfering with detection of the H3K27me3, as detected by an Alexa555 secondary antibody **A**. Residual Oil Red O in tissues post-processing for antibody detection was less prominent than on freshly sectioned slides (not shown); **B**. Overlay of H3K27me3 nuclei from panel **C** (Blue) onto the Oil Red O image from panel A. Note the absence of fluorescence from the most prominent Oil Red O deposits; C. Fluorescence microscopy image of anti-H3K27me3 detected with Alexa555 conjugated secondary antibody using filtering for Texas Red. Although fluorescence, apparently from nuclei, is not always spatially separated from Oil Red O deposits, the residual Oil Red O deposits are not contributing fluorescence in our processed tissues.
